# Effect of Geography and Captivity on Scat Bacterial Communities in the Imperiled Channel Island Fox

**DOI:** 10.3389/fmicb.2021.748323

**Published:** 2021-12-01

**Authors:** Nicole E. Adams, Madeleine A. Becker, Suzanne Edmands

**Affiliations:** Department of Biological Sciences, University of Southern California, Los Angeles, CA, United States

**Keywords:** 16S rRNA gene, captivity, Channel Island fox, conservation, microbiota

## Abstract

With developing understanding that host-associated microbiota play significant roles in individual health and fitness, taking an interdisciplinary approach combining microbiome research with conservation science is increasingly favored. Here we establish the scat microbiome of the imperiled Channel Island fox (*Urocyon littoralis*) and examine the effects of geography and captivity on the variation in bacterial communities. Using high throughput 16S rRNA gene amplicon sequencing, we discovered distinct bacterial communities in each island fox subspecies. Weight, timing of the sample collection, and sex contributed to the geographic patterns. We uncovered significant taxonomic differences and an overall decrease in bacterial diversity in captive versus wild foxes. Understanding the drivers of microbial variation in this system provides a valuable lens through which to evaluate the health and conservation of these genetically depauperate foxes. The island-specific bacterial community baselines established in this study can make monitoring island fox health easier and understanding the implications of inter-island translocation clearer. The decrease in bacterial diversity within captive foxes could lead to losses in the functional services normally provided by commensal microbes and suggests that zoos and captive breeding programs would benefit from maintaining microbial diversity.

## Introduction

The gut microbiome plays an essential role in the health and fitness of its host (Mcfall-Ngai et al., 2013; McKenney et al., 2018). As such, it is becoming increasingly clear that to fully understand a host one must examine their associated microbiome and the forces driving variation among microbial communities. This approach is burgeoning in the field of conservation biology, where anthropogenic effects such as habitat fragmentation (Barelli et al., 2015) and increasing temperatures (Fontaine et al., 2018) have been shown to alter host-associated microbial communities. Integrating microbiome research into conservation science is beneficial at multiple levels from health monitoring (Kreisinger et al., 2015; Bragg et al., 2020) to microbiome transplants in captive individuals (Trevelline et al., 2019; Guo et al., 2020). Therefore, characterizing the current bacterial communities in endangered host species is crucial to identify future changes in microbial diversity.

Here we investigated the gut microbiome of the Channel Island fox, *Urocyon littoralis*, a charismatic species that was recently rescued from the brink of local extinction and that still faces continuing threats (Coonan et al., 2010). *U. littoralis* is a dwarfed insular canid with a distinct subspecies on six of the Channel Islands off the coast of southern California, United States (Coonan et al., 2010). Formerly endangered, four of the six inhabited islands went through extensive population crashes losing upward of 90–96% of foxes, but have since recovered in population size (Coonan et al., 2010). Only one subspecies is still federally listed as threatened but all island fox populations face continued threats of human activity and climate change, including introduced animals, novel pathogens, and worsening prolonged droughts (Doak et al., 2013; Gould and Andelt, 2013; Gustafson, 2020; Sanchez and Hudgens, 2020; Bakker et al., 2021). With low genetic diversity within populations (Robinson et al., 2016), the microbiome may be particularly important in disease identification and protection. In fact, changes in the microbiome have been linked to mange and ear tumors in *U. littoralis* (DeCandia et al., 2019, 2020). Potential differences in microbiomes between populations (signifying underlying adaptive differences) could have profound conservation implications considering proposed inter-island translocation of foxes (Funk et al., 2016).

In addition to being of conservation concern, this system presents a natural experiment to test the effect of geography on the gut microbiome because the foxes originated from one mainland source, and migrated to the other islands in a known pattern with little further movement between islands (Hofman et al., 2016). Variation in the size and topography of the islands cause differences in temperature, precipitation, and wind (Schoenherr et al., 2003) and likely has contributed to the divergence of the gut microbiome. These geographically differing variables, as well as differences in anthropogenic impacts, across the islands influence the diet (Cypher et al., 2011, 2014) and pathogen prevalence (Clifford et al., 2006; Harris et al., 2013; Gaffney et al., 2015; Vickers et al., 2015) across fox populations. Knowing that diet and the immune system affect the gut microbiome, this system provides the opportunity to identify a potential correlation between geography and gut bacterial communities.

Broadly, geographic isolation of populations can lead to divergence in microbiota over time due to drift and/or local adaptation. While geography has been shown to play a role in shaping the gut microbiome in a number of taxa (Linnenbrink et al., 2013; Moeller et al., 2017; Grieneisen et al., 2019; Colborn et al., 2020), few studies have looked at these effects in intraspecific island populations. Relative to interspecific comparisons, intraspecific comparisons can be expected to minimize differences in host factors such as physiology, behavior and ecology, and therefore allow better resolution of factors such as host sex and condition, as well as the effects of geography. Here we investigate a species with substantial geographic subdivision, allowing a strong test of the correspondence between geography and microbial variation.

Captivity, often a tool in conservation management, has also been shown to alter gut microbial diversity (Villers et al., 2008; Kohl et al., 2014; Cheng et al., 2015; Clayton et al., 2016; McKenzie et al., 2017; Wasimuddin et al., 2017). Conditions in captivity are markedly different than those in the wild including changes in diet, interspecific interactions, and health care, any of which can impact microbial community makeup. Across multiple taxa, studies have shown a decrease in microbial diversity in captive mammals compared to wild individuals (Kohl et al., 2014; Cheng et al., 2015; Clayton et al., 2016; McKenzie et al., 2017; Wasimuddin et al., 2017), which could impact individual health and/or release success (Wienemann et al., 2011). However, a recent study showed that the microbiome of Tasmanian devils was restored upon release in the wild (Chong et al., 2019). The effects of captivity on the microbial community appear to affect diet specialists more than generalists (Kohl et al., 2014) and carnivores more than herbivores (McKenzie et al., 2017). Currently, there are island foxes in multiple mainland zoos, and previously four wild populations went through captive breeding programs, yet it remains unknown how captivity affects the microbiome of *U. littoralis*.

The objective of this study is to characterize the bacterial communities in island fox scat and to test for associations between geography and bacterial composition. We also compare natural populations to captive populations. We predict that geography and captivity will correlate with shifts in bacterial community composition. For the effects of captivity our predictions are twofold: (1) we predict that bacterial diversity will decrease in captive foxes compared to their wild counterparts, as shown in other canids (McKenzie et al., 2017) and (2) we predict that bacterial communities from captive foxes will be more similar to other captive foxes than they are to those from the host source population. Understanding the drivers of the microbiome will provide additional tools for the conservation of these threatened animals and potentially provide a minimally invasive way to gauge health by relating microbial community profiles with fox health status.

## Materials and Methods

### Sampling and DNA Extraction

**FIGURE 1:**
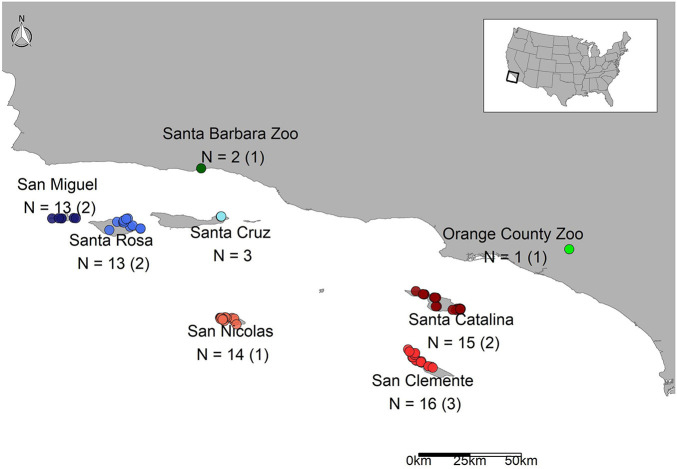
Map of scat samples across the Channel Islands and the mainland. The sample sizes are listed followed by the number of replicates, which are listed in parentheses.

Scat samples were collected during annual trap monitoring of *U. littoralis* during the fall and winter of 2014–2015 by managers or contractors on the Channel Islands off the coast of southern California, United States (San Miguel Island, SMI; Santa Rosa Island, SRI; Santa Cruz Island, SCZ; Santa Catalina Island, CAT; San Clemente Island, SCL; San Nicolas Island, SNI; Figure 1). See Supplementary Table 1 for sample details. Samples were collected from traps of individual foxes, so the identity of the foxes were known. Scat samples from captive *U. littoralis* were collected opportunistically in 2014 (Orange County Zoo, OCZ; Santa Barbara Zoo, SBZ). Scat samples were collected in ethanol and then stored at −80°C until DNA extraction could be performed. Total genomic DNA was extracted from the scat samples using the E.Z.N.A Genomic DNA Isolation Kit for stool samples (Omega Bio-Tek, Norcross, GA, United States) following the manufacturer’s instructions with minor changes. Modifications to the protocol included incubating with Proteinase K for 20 min (an increase of 10 min) and eluting the DNA with 100 μL of elution buffer. Glass beads were used during the extractions to ensure bacteria were lysed, per the extraction kit’s instructions. Each batch was extracted alongside an experimental blank containing molecular grade water instead of sample to monitor potential contamination, and samples were extracted no more than 8 months after being collected in the field. Genomic DNA was quantified using an Invitrogen Qubit 2.0 Fluorometer (Carlsbad, CA, United States).

### Library Preparation and Sequencing

The V4 hypervariable region of the 16S rRNA gene was amplified from DNA extracted from *U. littoralis* scat using bacterial “universal” primers 515F and 806R, to which 4 ambiguous bases and variable barcodes on the forward primer and variable indexes on the reverse primers were added (Supplementary Table 2; Bartram et al., 2011; Caporaso et al., 2011). We followed a modified Earth Microbiome Project protocol (Gilbert et al., 2014). Briefly, the 16S V4 region was amplified in triplicate reactions using Phusion High-Fidelity DNA Polymerase (New England Biolabs, Ipswich, MA, United States) and then the reactions were cleaned with AMPure XP beads (Beckman Coulter, Brea, CA, United States) at a 1:1 ratio of beads to PCR product volume. The amplicon quantity was again evaluated with the Qubit and the quality was further evaluated with a Bioanalyzer analysis (Agilent Technologies, Santa Clara, CA, United States). Finally, samples were pooled equimolarly for sequencing. We sequenced 95 samples (Supplementary Table 1) as 300 bp paired-end reads on an Illumina MiSeq at UCLA’s Genotyping Core (Los Angeles, CA, United States). Blank samples (*N* = 5), technical replicates (repeat sequencing library preparations on the same DNA extraction; *N* = 10), and a positive control (an even mock community) were sequenced as part of the 95 samples. For the Orange County Zoo samples (sample 1 = OCZ.1a and OCZ.1b) and one Santa Barbara Zoo sample (sample 102003 = SBZ.2a and SBZ.2b) biological replicates were sequenced (meaning two different scat samples from the same organism). These two zoo individuals (SBZ.2a/b and OCZ.1a/b) were born in captivity but are descended from San Clemente Island foxes. One zoo sample (SBZ.1) was born on San Clemente Island but was brought into captivity.

### Sequence Processing

Leading ambiguous bases were trimmed from forward reads with Trimmomatic v. 0.33 (Bolger et al., 2014). Barcodes were extracted with extract_barcodes.py, and samples were demultiplexed in QIIME1 v. 1.9.1 (Caporaso et al., 2010). Demultiplexed reads were merged into one artifact with QIIME1 and then imported back into QIIME2. Sequences were processed using denoise-paired in the DADA2 v. 2018.6.0 plugin (Callahan et al., 2016) in QIIME2. Processing included the following steps with default settings unless otherwise noted: denoising, trimming primers (–p-trim-left-f 19, –p-trim-left-r 20), removing chimeras and dereplicate reads, trimming forward reads to a minimum of 270 bp and reverse reads to a minimum of 210 bp based on quality scores, filtering out targets below those length cutoffs, and inferring amplicon sequence variants (ASVs) for both forward and reverse reads. Non-clustering methods that generate sequence variants are convincingly argued to be preferred over traditional 97% sequence clustering approaches due to their higher resolution and reproducibility (Callahan et al., 2017). Taxonomy was assigned using feature-classifier classify-sklearn (Pedregosa et al., 2011) in QIIME2 using the Greengenes database v. 13-8 (DeSantis et al., 2006), then non-target ASVs (mitochondria, archaea, and chloroplasts) were removed. The mock community sample was removed from the dataset for analysis.

### Decontamination, Filtering, and Transformation

The data were imported into RStudio v. 1.1.456 (RStudio Team, 2018) with R v. 3.5.1 (R Core Team, 2016) using the Qiime2r v. 0.99.1 package (Bisanz, 2018) creating an object to use with the Bioconductor R package Phyloseq v. 1.24.2 (McMurdie and Holmes, 2013). Extraction blanks were addressed first by identifying potential contaminants using Decontam v. 1.0.0 (Davis et al., 2018). We used the “combined” method, which is based on frequency (variation based on DNA concentration) and prevalence (increased prevalence in negative controls) probabilities in tandem with a Fisher’s combined probabilities test. This method identified 12 ASVs as potential contaminants which were then removed from the dataset (Supplementary Table 3). After decontamination, extraction blanks were also removed from the dataset. Then technical replicates were merged using mean counts in Phyloseq [see Supplementary Figure 1 for principal coordinates analysis (PCoA) of technical replicates]. The ASV count table was transformed using a variance stabilizing transformation (Anders and Huber, 2010; McMurdie and Holmes, 2014) in DESeq2 v. 1.20.0 (type = “poscounts” to estimate size factors; Love et al., 2014) for beta diversity assessment. For use in Phyloseq (McMurdie and Holmes, 2013), negative transformed values were set to zero as advised by the authors^1^.

### General Taxonomy and Beta Diversity Visualization

The dominant taxa present in the scat bacterial microbiome were identified based on ASV proportions (not transformed), and differences among sources were evaluated with analysis of variance (ANOVA) and Tukey HSD *post hoc* tests. First we agglomerated the count table at the phylum level for all phyla except for the Proteobacteria phylum which was split into its major classes for analysis due to its complexity (Hug et al., 2016), and phyla representing less than 5% of the ASVs were grouped together as “other.” With similar methods, we looked at the top genera with the largest proportions. To address beta diversity, a Bray–Curtis dissimilarity matrix was calculated on variance-stabilizing transformed data and used with hclust (method = ward.D2) to generate a hierarchical clustering dendrogram. The transformed data were also used to conduct a principal coordinates analysis (PCoA) based on Bray–Curtis dissimilarities with Phyloseq.

### Testing for Group Differences and Differential Abundance

Testing for statistical differences in scat bacterial communities was done on the transformed ASV counts with Vegan v. 2.5-3 (Oksanen et al., 2020). Permutational ANOVAs were calculated using adonis (permutations = 99999) based on Bray–Curtis dissimilarity (Anderson, 2006, 2017) only on samples with no missing data (Supplementary Table 4A). The data were transformed separately for each dataset after samples with missing data were removed. We did this across all samples and then again for each island and the captive versus wild dataset. The assumption of homogeneity in dispersion within groups was tested with betadisper. We evaluated the best models by including all variables regardless of dispersion and then we sequentially removed variables with low variance explained (*R*^2^). The models were evaluated with Akaike information criterion (AIC), taking the lowest value as the best model that was used in the permutational ANOVA. The source code for the AIC method is available at Kdyson (2020). For the combined dataset that included all islands, we tested the effects of age, body condition, month and year collected, sex, source (each island or zoo), weight, and the extraction group in which the sample belonged. For each island, we evaluated the same variables excluding source and year collected when it did not vary from month collected (SNI). For the captive dataset, we included the following variables: captivity status, age, month and year collected, sex, and if the individual was born in captivity or born on an island then taken into captivity. Body condition is a common measurement scale used to gauge animal health (Coonan et al., 2014) that goes from emaciated (body condition = 1) to obese (body condition = 5) and was recorded by wildlife managers in the field. We then examined differential abundances in ASVs for comparisons with significant permutational ANOVA results using the R package DESeq2 and a significance level of 0.01 (with the input being the untransformed, but filtered, count table). We also looked at differentially abundant genera by first agglomerating the count table at the genus level. We looked at alpha diversity in comparisons with significant permutational ANOVA results by calculating Chao1 richness index and Shannon diversity index using untransformed count tables and plot_richness in Phyloseq to understand relative differences.

## Results

### Taxonomy

**FIGURE 2:**
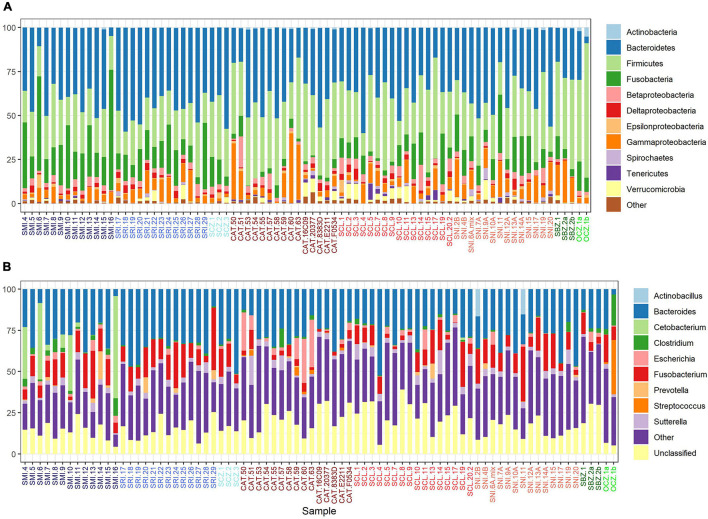
Percent relative abundance of the major taxa **(A)** and genera **(B)** recovered from *U. littoralis* scat per sample. Sample names are colored by source (island or zoo).

Bacteroidetes, Firmicutes, Fusobacteria, and Proteobacteria (classes Gammaproteobacteria and Betaproteobacteria) were the dominant phyla in the scat samples; the first two accounting for over 67% of the recovered sequences and the top 6 major taxa collectively accounting for about 94% of the total sequences in each group (Figure 2A). Seven major taxa were significantly different between sources. Firmicutes had the highest number of significant pairwise comparisons and was mainly elevated in zoo foxes compared to island foxes (Supplementary Table 5A and Supplementary Figure 2). Foxes from SMI and SNI were significantly different between 3 out of 7 major taxa, the most of any pairwise comparison (Supplementary Table 5A). Largely foxes from SMI had significantly elevated proportions of Fusobacteria (Supplementary Table 5A and Supplementary Figure 2). Bacteroidia made up the largest average proportion (37.8 ± 12.2%; mean ± 1SD) of identified classes followed by Clostridia (27.4 ± 10.0%). The same pattern held for order; Bacteroidales and Clostridiales made up the largest represented orders at comparable percentages to their respective classes. The top genera were Bacteroides and Fusobacterium accounting for over 38% of the recovered sequences (Supplementary Table 5B and Figure 2B). Of the top genera, 6 were significantly different between sources. *Escherichia* was elevated in foxes from CAT compared to SRI and the other southern islands of SCL and SNI. Most notably, however, *Clostridium* and *Streptococcus* were significantly elevated in captive foxes from OCZ compared to every other source (Supplementary Table 5B and Figure 2B).

### Between-Group Diversity Visualization

**FIGURE 3:**
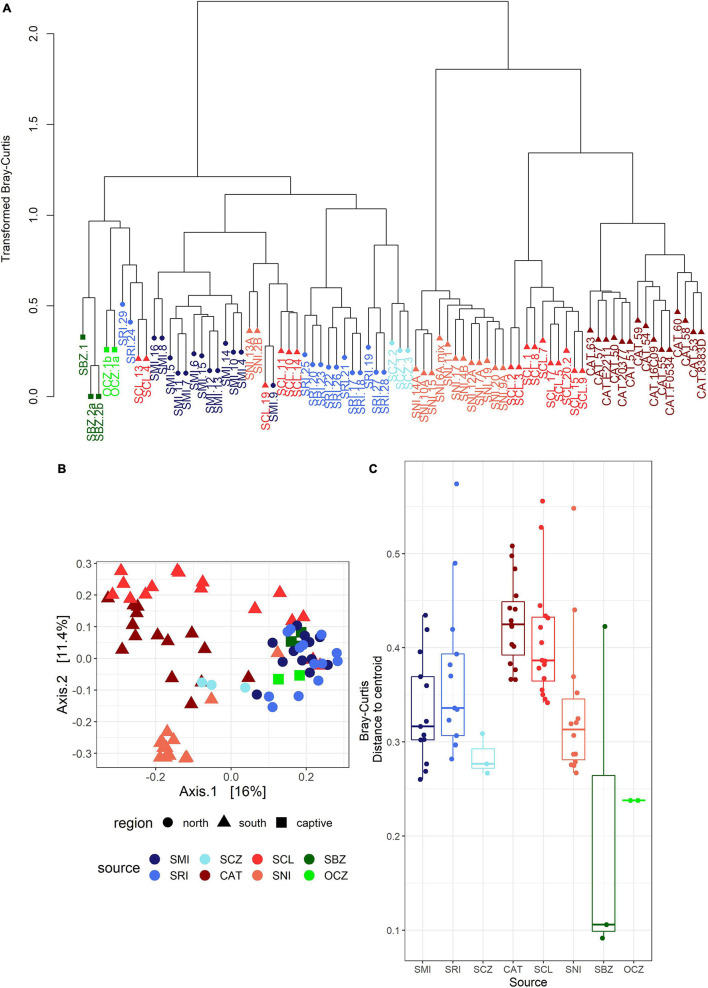
**(A)** Dendrogram of transformed data based on Bray–Curtis dissimilarity. Sample names are colored by island or zoo (see Figure 1) and shapes correspond to region: circle = northern islands, triangle = southern islands, and square = captive samples. **(B)** Principal coordinates analysis (PCoA) based on Bray–Curtis dissimilarity. The points are samples colored by source (island or zoo) and the shapes represent northern or southern Channel Islands and captive samples. **(C)** Boxplot of multivariate dispersion as measured by the distance to the centroid per sample and displayed by source.

The bacterial communities largely clustered into two main groups based on geography (Figures 3A,B). We identified two main clusters based on the dendrogram (Figure 3A). One cluster is entirely composed of samples from the southern islands (SNI, SCL, and CAT), while the other contained all of the northern and zoo samples, as well as a subset of southern samples including about half of the SCL samples and two from SNI. In the PCoA (Figure 3B), axis 1 (16%) largely described the distance between the northern/zoo samples group and the southern islands group, including the aforementioned exceptions, and axis 2 (11.4%) predominantly described the variation within those groups.

### Drivers of the Bacterial Communities

The strongest driver of differences among bacterial communities was source (Table 1 and Figure 3C). Across all samples, weight and month of sample collection also significantly influenced the bacterial communities (Table 1). Although, it is important to note that there is a known significant difference in body size among the islands (Wayne et al., 1991), which was also found in our weight data (ANOVA_source_, *P* = 5.0 × 10^–9^). The highest mean weight was found on CAT, and SNI had the lowest mean weight. When broken out by island, month was the driving factor on SRI, sex the largest factor on CAT, and on SCL weight and year drove the difference in bacterial communities (Table 1 and Supplementary Figure 3). To look further into the influence of collection timing on SRI and SCL, we evaluated precipitation over the period assessed for each island (Supplementary Figure 4). Sex was also found to be the driving factor on SNI; however, the pattern was driven by one male, and when removed there were no significant factors (Table 1 and Supplementary Figure 3). We investigated the difference between the southern samples that grouped with the northern samples and those that did not and found the best model included weight, month collected, and the group in which the sample was extracted as significant drivers of bacterial community difference. For results from our model testing see Supplementary Tables 4B–H. It should be noted that not all variables were homogenously distributed, but the heterogeneity in the source term is likely biologically relevant (Supplementary Table 6 and Figure 3C). Further, SCZ did not have enough samples (*N* = 3) to conduct the permutational ANOVA, and there were no significant factors identified for SMI likely due to small sample size (*N* = 6) after removing samples with missing data.

**TABLE 1:** Results from the permutational ANOVAs (Bray–Curtis dissimilarity, permutations = 99999) for the best models.

	**Df**	**sumSq**	**meanSq**	***F***	***R* ^2^**	***P***	**AIC**
**Islands (*N* = 62)**							149.4442
Source	5	5	0.93	6.1	0.340	**0.00001**	
Month collected	4	1	0.24	1.6	0.070	**0.00224**	
Weight	1	0	0.35	2.3	0.025	**0.00426**	
Residuals	51	8	0.15	–	0.570	–	
Total	61	14	–	–	1	–	
**SMI (*N* = 6)**							−4.0375
Age	1	0	0.11	1.10	0.19	0.35	
Year collected	1	0	0.11	1.10	0.19	0.401	
Weight	1	0	0.11	1.10	0.20	0.338	
Extracted group	1	0	0.14	1.40	0.25	0.101	
Residuals	1	0	0.10	NA	0.17	–	
Total	5	1	–	–	1	–	
**SRI (*N* = 13)**							9.3148
Month collected	1	0	0.35	2.60	0.190	**0.00367**	
Residuals	11	2	0.14	–	0.810	–	
Total	12	2	–	–	1	–	
**CAT (*N* = 15)**							17.2776
Sex	1	0	0.33	1.80	0.120	**0.0175**	
Residuals	13	2	0.19	–	0.88	–	
Total	14	3	–	–	1	–	
**SCL (*N* = 15)**							14.4247
Year collected	1	0	0.39	2.70	0.150	**0.0155**	
weight	1	0	0.43	2.90	0.170	**0.00777**	
Residuals	12	2	0.15	–	0.680	–	
Total	14	3	–	–	1	–	
**SNI (*N* = 10)**							1.8308
Sex	1	0	0.31	3.1	0.270	**0.0239**	
Condition	1	0	0.11	1.2	0.099	0.3060	
Age	1	0	0.10	1.1	0.091	0.3380	
Month collected	1	0	0.12	1.2	0.100	0.2160	
Weight	1	0	0.10	1.0	0.087	0.4290	
Extracted group	1	0	0.10	1.0	0.090	0.3900	
Residuals	3	0	0.10	NA	0.260	–	
Total	9	1	–	–	1	–	
**SNI (females, *N* = 9)**							0.9833
Month collected	1	0	0.12	1.20	0.14	0.195	
Residuals	7	1	0.10	–	0.86	–	
Total	8	1	–	–	1	–	
**Captivity (*N* = 21)**							28.01
Wild vs. captive	1	1	0.89	6.2	0.210	**0.00001**	
Month collected	3	1	0.30	2.1	0.210	**0.00375**	
Year collected	1	0	0.41	2.9	0.094	**0.00757**	
Residuals	15	2	0.14	NA	0.490	–	
Total	20	4	–	–	1.00	–	

*P-values for significant factors are in bold. Df, degrees of freedom; sumSq, sum of squares; meanSq, mean squares; F, F-statistic; R^2^, variance explained; P, p-value; AIC, Akaike information criterion.*

We found 173 differentially abundant ASVs significant at the α = 0.01 level in our significant factors across islands. On SRI we found 72% of the significantly differentially abundant ASVs were elevated in samples collected in November, while 28% of the ASVs were elevated in December (Table 2). On CAT we found 38% of the significantly differentially abundant ASVs were elevated in female samples, opposed to 62% of the ASVs being elevated in males (Table 2). Additionally, on SCL we found the majority of the significantly differentially abundant ASVs were elevated in samples collected from 2014 (87%) and heavier foxes (88%). Since weight was evaluated as a continuous variable, this result can be interpreted as an increase in ASV abundance with increasing weight. Both on SRI and SCL the genus *Megamonas* was significantly differentially abundant in samples collected in December and January (for additional differences at the genus level see Supplementary Table 7).

**TABLE 2:** Top differentially abundant ASVs for significant comparisons.

**Comparison**	**ASV**	**log2Fold change**	**lfcSE**	**padj**	**Mean relative abundance**	**Lowest classification**	**Blast hit**	**Accession**	**Identity (%)**	**Score**
**SRI**										
Nov (33)	79b4767bedcb8bbb5 69be837c0e91fc9	27.7	3.1	6.17E-17	0.008	*Rickettsiella*	*Diplorickettsia massiliensis* 20B	NR_117407.1	98.01	431
Dec (13)	e42382ea3fa11490 94d507e3167e462b	−28.7	3.01	7.72E-19	0.016	Enterobacteriaceae	*Rosenbergiella australiborealis* strain CdVSA 20.1	NR_126305.1	100	453
**CAT**										
Female (19)	290aac7b5cfcb9 e70ca98c874f099965	26	2.99	3.83E-15	0.004	*Fusobacterium*	*Fusobacterium mortiferum* strain DSM 19809	NR_117734.1	98.8	448
Male (31)	b15f5845058247119 ae560a6b19e2e4d	−25.5	2.98	6.80E-15	0.001	*Faecalibacterium prausnitzii*	*Faecalibacterium prausnitzii* strain ATCC 27768	NR_028961.1	98.41	442
**SCL**										
2014 (46)	13173fa1a3d63abc7 f566a5a4cbf27bc	25.1	3.42	1.43E-11	0.003	*Sutterella*	*Parasutterella secunda* strain JCM 16078	NR_113328.1	95.63	403
2015 (7)	3c994ad0917947698 cf5235a951c4df0	−28.9	3.32	1.79E-15	0.007	*Bacteroides*	*Bacteroides coprocola* DSM 17136 strain M16	NR_041278.1	98.41	442
Low weight (3)	06c214f56026f8ffd 260f153bd0cf856	19.8	5.11	0.00637	0.005	*Prevotella*	*Prevotellamassilia timonensis* strain Marseille-P2831	NR_144750.1	93.63	375
High weight (21)	f62358a1dcad765 3dddf044a1b7277c3	−23.7	5.22	0.00212	3.5E-6	*Anaerofilum*	*Anaerofilum pentosovorans* strain Fae	NR_029313.1	94.8	390
**Captivity**										
Wild (147)	845be19afef094e468 6bb348b963a973	28.0	2.83	3.36E-21	0.005	*Brassicaceae*	*Ruficoccus amylovorans* strain CC-MHH0563	NR_156844.1	88.49	303
Captive (34)	386622a3b30e3e131 e44a1db5038508d	−27.9	3.43	9.25E-15	0.006	*Streptococcus luteciae*	*Streptococcus alactolyticus* strain ATCC 43077	NR_041781.1	100	464
2014 (38)	0dc4d1fbaf41217 1d94cf201be277c26	26.1	2.68	2.65E-19	7.4E-4	*Campylobacter*	*Campylobacter upsaliensis* strain NCTC 11541	NR_118528.1	100	453
2015 (36)	3c994ad0917947698 cf5235a951c4df0	−28.8	3.11	1.2E-17	0	*Bacteroides*	*Bacteroides coprocola* DSM 17136 strain M16	NR_041278.1	98.4	435
Early months (30)	e60001b105ddb39108 7c1f6d34af54c9	3.2	0.33	4.16E-22	0	Ruminococcaceae	*Eubacterium coprostanoligenes* strain HL	NR_104907.1	94.8	395
Late months (85)	efcd0461ac1ed0fea 763be1234b924d9	−12.8	0.32	0	0.007	*Megamonas*	*Megamonas funiformis* YIT 11815	NR_041590.1	100	453

*The values in parentheses are the total number of differentially abundant ASVs at a significance level of α = 0.01 for that comparison. Adjusted p-values are reported from a Wald test. Top Blast hits were found using the 16S database in NCBI and reporting the top hit with the highest score. LfcSE indicates the standard error of the logarithmic fold change, and the lowest classification is from the taxonomic assignment based on the Greengenes database.*

**FIGURE 4:**
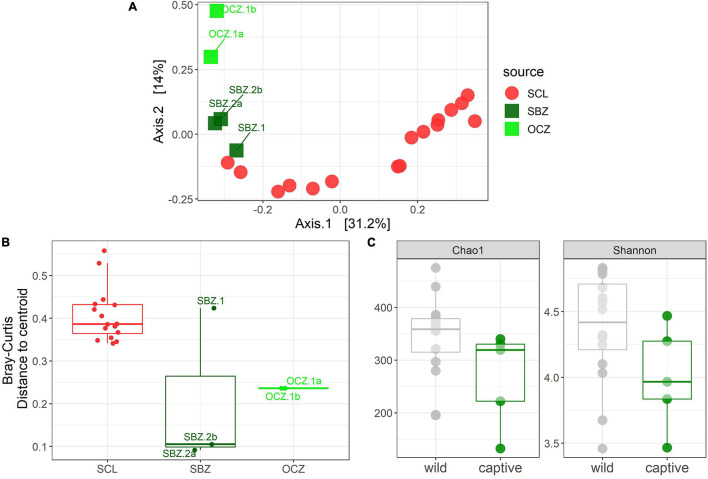
**(A)** Principal coordinates analysis (PCoA) based on Bray–Curtis dissimilarity of samples from SCL and captivity (SBZ, OCZ). **(B)** Boxplot of multivariate dispersion as measured by the distance to the centroid per sample and displayed by source. **(C)** Chao 1 and Shannon diversity metrics between wild and captive samples.

To explicitly test for the effect of captivity on the scat bacterial microbiome, we probed differences between the captive and SCL samples because all zoo individuals were either descendants of SCL foxes or born on the island and then taken into captivity (Figure 4). We found that the best model for these data included the captivity factor along with the month and year that the sample was collected, and all three terms were significant (Table 1). Body condition and weight could not be considered due to missing data. We found that the majority of the significantly differentially abundant ASVs were elevated in wild samples (81%) compared to captive (Table 2). There was a nearly equal number of significantly differentially abundant ASVs between sampled years, but more differentiated ASVs from samples collected in months later in the year. In fact, *Megamonas* was again seen as a significant player in samples from later months, which could be driven by the same result found in SCL samples alone (Supplementary Table 7).

### Patterns of Alpha Diversity

Statistical analyses on alpha diversity can be misleading (Willis, 2019), therefore we explore qualitative alpha diversity patterns to gain insight into the statistically significant factors we identified through the permutational multivariate analysis of variance (PERMANOVAs). When comparing across sources, scat bacterial communities from the three southern islands, especially CAT, generally had higher ASV richness and evenness compared to the northern Channel Islands (Supplementary Figure 5A). Across all samples analyzed, ASV richness and evenness peaked in October, while richness, but not evenness, trended toward increasing with weight (Supplementary Figures 5B,C). In bacterial communities from SRI, scat collected in November had higher richness and evenness compared to those collected in December (Supplementary Figure 5D). Males had higher evenness, but not richness, compared to females on CAT (Supplementary Figure 5E). Meanwhile, the evenness of bacterial communities on SCL declined with increasing weight while richness was constant (Supplementary Figure 5F). Both richness and evenness were higher in samples collected in 2014 compared to those collected in 2015 on SCL (Supplementary Figure 5G). Samples collected from captive foxes had lower richness and evenness than those collected from wild foxes (Figure 4C).

## Discussion

Recent advances and cost efficiency in high-throughput sequencing has made culture-independent methods of surveying microbial communities tractable. Combined with the insight that host-associated microbiota may aid in rapid adaptation to environmental change (Alberdi et al., 2016), conservation biologists are increasingly using sequencing to assess microbiome diversity. We sought to characterize a baseline and identify determining factors in each isolated subspecies within the threatened *U. littoralis* by using high-throughput amplicon sequencing. Few studies address microbiome differences among geographically isolated intraspecific populations, yet such studies provide insight into the mechanisms driving the composition of commensal microbes. We report distinct bacterial communities on each island as well as between wild and captive foxes.

### Geography Drives the Island Fox Microbiome

Geographic source is a large contributor to bacterial community composition in *U. littoralis* scat (Figure 3). This result is expected, given that many biotic and abiotic factors change with biogeography which have been previously shown to affect the bacterial microbiome (Linnenbrink et al., 2013). Overall, the scat bacterial communities of *U. littoralis* were made up of the usual mammalian gut taxa; Bacteroidetes, Firmicutes, Fusobacteria, and Proteobacteria are consistent, abundant players in the canid scat bacterial communities (Ley et al., 2008; Zhang and Chen, 2010). In other studies, bacterial community differences among populations have been shown to be driven by differences in diet (Barelli et al., 2015), which could also contribute to the differences we see across islands in our dataset. For example, the lower relative abundance of Firmicutes classes Bacilli and Clostridia on SMI and SRI, and the higher abundance of Fusobacteriales, compared to the southern islands could reflect a more protein-rich diet (Deitch et al., 1987; Hang et al., 2012; Finlayson-Trick et al., 2017). This hypothesis is consistent with data showing that foxes on SRI consume the lowest amount of fruit among the islands and that on SMI the only fruit consumed by foxes is that of a non-native plant and instead they rely heavily on insects (Cypher et al., 2014). The month the sample was collected likely reflects differences in diet as well, as discussed later in this section. Interestingly, we found a unidirectional pattern in which a subset of samples from southern islands grouped with northern samples, but none of our predictor variables were able to explain this pattern (data not shown). More work is therefore needed to understand this finding and other deviations from a previous phylogenetic tree (Robinson et al., 2016). In future work we will therefore conduct a formal test of phylosymbiosis to better understand the impact of host genetics on the microbiome.

While geography had the greatest impact on the scat bacterial communities, weight, timing of the collection, and sex also contributed to the geographic patterns. Weight was a significant factor that influenced the bacterial community within and among islands, although as previously noted body size (including weight) is significantly different among islands (Wayne et al., 1991). Additionally, weight was a significant factor within an island, specifically on SCL, where the majority of the significantly differentially abundant ASVs in heavier samples were associated with Clostridia. This result is concordant with previous work that showed Clostridiales to be more abundant in obese pet dogs (Handl et al., 2013). Obesity has also been associated with increased levels of Firmicutes (Ley et al., 2005, 2006; Turnbaugh et al., 2009; Riva et al., 2017), and while foxes are rarely overweight especially in the wild, we found a similar pattern of increased amounts of Firmicutes in heavier foxes. Weight is a well-studied factor, especially in humans and mice, that affects the microbiome (Bäckhed et al., 2004; Ley et al., 2005, 2006; Riva et al., 2017), and thus also an expected result of our study.

The timing of sample collection also significantly influenced the scat bacterial communities in *U. littoralis* both among and within islands, specifically on SCL and SRI. The top NCBI Blast hit for the ASV with the largest effect (largest log2 fold change) in foxes in 2014 on SCL was *Parasutterella secunda*, which is proposed to be a core taxon that has a role in bile acid maintenance and cholesterol metabolism (Ju et al., 2019). The top NCBI Blast hit for the ASV with the largest effect in foxes in 2015 on SCL was *Bacteroides coprocola*, which is also associated with bile and the breakdown of plant polysaccharides (Kitahara et al., 2005). At the genus level *Megamonas*, shown to ferment glucose to short chain fatty acids (Chevrot et al., 2008; Sakon et al., 2008), consistently came up associated with collection timing. Temporal changes in the gut microbiome can be associated with seasonal changes in diet (Ren et al., 2017). The winter is typically the rainy season in southern California, which drastically changes the flora and fauna for the subsequent months. Exemplary of this process, the total rainfall on SRI and SCL in December of 2014 was 9–25 times greater than the surrounding months [Supplementary Figure 4; (Western Regional Climate Center, 2019)] and likely changed food source availability for the foxes. The top NCBI Blast hit for the ASV with the largest effect in foxes in November on SRI was *Diplorickettsia massiliensis*, which is an obligate bacterium of arthropods (Mediannikov et al., 2010). The top NCBI Blast hit for the ASV with the largest effect in foxes in December on SRI was *Rosenbergiella australiborealis*, which was isolated from plant nectar (Lenaerts et al., 2014). The taxa that correlate with collection timing, especially on SRI, appear to be more associated with environmental drivers of diet than with host metabolism.

Sex contributed to the differences in bacterial communities, which is consistent with several mammalian gut studies (McKenna et al., 2008; Phillips et al., 2012; Nelson et al., 2013; Menke et al., 2017). While this result was significant on two islands, the pattern was likely driven by one male sample on SNI so we focused on the differences found between sexes in CAT individuals. The top differentiated bacteria between sexes appear to be associated with diet. The top NCBI Blast hit for the ASV with the largest effect in females was *Fusobacterium mortiferum*, which metabolizes sucrose (Pikis et al., 2002). The top NCBI Blast hit for the ASV with the largest effect in males was *Faecalibacterium prausnitzii*, which is a well-studied gut commensal that produces butyrate and is indicated as a sentinel in gut health (Sokol et al., 2008; Khan et al., 2012). While sex-biased differences in diet can explain differences in gut microbiota (Bolnick et al., 2014), the previous study of the island fox’s diet did not look at differences between sexes (Cypher et al., 2014) so more work is needed here to tease apart that relationship. Alternatively, sex hormones have been proposed as a mechanism for sex-specific microbiome differences (Org et al., 2016).

### Captivity Alters the Fox Microbiome

Captivity significantly altered the scat microbiome of *U. littoralis* as evidenced by shifts in specific taxa (i.e., Firmicutes) and lower overall bacterial diversity. Despite the small sample sizes including biological replicates, this strong pattern emerged and is consistent with previous studies (Amato et al., 2013; Kohl et al., 2014; McKenzie et al., 2017; Guo et al., 2019; Schmidt et al., 2019). The top NCBI Blast hit for the ASV with the largest effect in wild foxes was *Ruficoccus amylovorans*, which is a diazotroph that reduces nitrate and degrades starch (Lin et al., 2017). In contrast, the top NCBI Blast hit for the ASV with the largest effect in captive foxes was *Streptococcus alactolyticus*, a potentially pathogenic species associated with diseases (ruminal acidosis, infective endocarditis, and colorectal cancer) in humans and animals (Jans et al., 2015; Jans and Boleij, 2018). Furthermore, several subspecies in the *Streptococcus bovis/Streptococcus equinus* Complex, to which *S. alactolyticus* belongs, have antimicrobial resistance (Jans and Boleij, 2018; Pompilio et al., 2019). While the mechanism of the bacterial shifts seen in this study remains unknown, previous studies have suggested changes in diet between captive individuals and their wild counterparts drive community differences in microbiota (Clayton et al., 2016; McKenzie et al., 2017; Metcalf et al., 2017; Greene et al., 2018; Oliveira et al., 2020). In the wild, island foxes are opportunistically omnivorous feeding on small mammals, insects, and native fruits (Cypher et al., 2014), while individuals in captivity are fed food items not found on the Channel Islands [e.g., dog food, hard-boiled eggs, fruits, and vegetables; (Coonan et al., 2010)]. Our results point to the influence that captivity can have on the fox immune system and subsequently the scat bacterial community. Reduced gut microbiome diversity can affect nutrient absorption and immune system function, ultimately affecting the overall health of the organism (Wienemann et al., 2011; Amato, 2013; Bragg et al., 2020). While there is some evidence that captive animals can gain back lost microbes upon release into the wild (Chong et al., 2019), carnivores have been shown to be more susceptible to losing microbial diversity in captivity (Metcalf et al., 2017) and there is evidence that it is more difficult to gain back wild microbes with an increasing number of generations in captivity (Webster et al., 2011; Becker et al., 2014). Going forward it will be imperative to manage host-associated microbiomes for captive foxes, especially if they will be released into the wild. To encourage a more diverse gut microbiome in captivity, managers could increase microbial reservoirs in enclosures (i.e., Loudon et al., 2014), shift diets to be more diverse and mimic natural food items, and decrease the use of antibiotics, as presented in Chong et al. (2019). More intensive approaches, including using prebiotics and probiotics that reflect the wild microbiome and/or fecal transplants could also be included to maintain diverse gut microbes.

For the captive samples, the PCoA (Figure 4A) and multivariate dispersion (Figure 4B) both show that SBZ2 and OCZ1 (both born in captivity) are similar to each other, while SCZ1 (born on SCL) is more similar to SCL. This pattern, while not confirmed statistically, suggests that inoculation from the natal environment may be more important than current environmental effects, which warrants further study. This legacy effect has been well-characterized in humans (Bokulich et al., 2016) and identified in other taxa (Jacob et al., 2015; Martínez-García et al., 2016; Gillingham et al., 2019). Some organisms have been able to maintain 60–70% of their wild microbes in captivity (Kohl and Dearing, 2014; Kohl et al., 2017), which could suggest that animals born in the wild but housed in captivity may have better health outcomes than those born in captivity.

### Conservation Implications

In this first assessment of the scat microbiome across all islands of the Channel Island fox, we found patterns largely driven by geography, with food availability emerging as a strong contributing factor. These results add to the mounting evidence of distinctions among the six subspecies. Discussions of a genetic rescue to augment island populations of low genetic diversity by introducing animals from islands of higher genetic diversity will have to consider the microbiome differences as they represent possible divergences in natural history (diet) and immune systems that could be incompatible with other island-specific pathogens (Kohl et al., 2018). Secondly, even though there is extremely low genome-wide diversity in *U. littoralis*, a recent study found no signs of inbreeding depression in skeletal morphological traits (Robinson et al., 2018). The prevailing thought is that, on some of the islands, foxes have been able to persist despite the negative consequences of small population sizes and low genetic diversity. Perhaps other factors, such as the microbiome, contribute to their longevity and health, and the plasticity of the microbiome contributes to acclimatizing and eventually adapting to demographic and environmental changes (Alberdi et al., 2016). The insights provided in this study attest to the potential that microbiome research has for informing conservation science.

## Data Availability Statement

The dataset generated for this study can be found in the NCBI SRA (BioProject PRJNA560412). The R code and metadata for the analyses are available on GitHub https://github.com/NicoleAdams-sci/islandFox_microbiome.

## Ethics Statement

Ethical review and approval was not required for the animal study because we did not conduct the field work. Instead, trained personnel from other institutions with appropriate permits collected the scat samples and later made them available for this study (see section “Acknowledgments”).

## Author Contributions

SE and NA conceived and designed the study. NA and MB performed the laboratory work and analyzed the data. NA and SE wrote the manuscript. All authors contributed to the article and approved the submitted version.

## Conflict of Interest

The authors declare that the research was conducted in the absence of any commercial or financial relationships that could be construed as a potential conflict of interest.

## Publisher’s Note

All claims expressed in this article are solely those of the authors and do not necessarily represent those of their affiliated organizations, or those of the publisher, the editors and the reviewers. Any product that may be evaluated in this article, or claim that may be made by its manufacturer, is not guaranteed or endorsed by the publisher.

## Footnotes

1
http://bioconductor.org/packages/devel/bioc/vignettes/phyloseq/inst/doc/phyloseq-FAQ.html

## Abbreviations

ANOVA: analysis of variance
ASV: amplicon sequencing variant
CAT: Santa Catalina Island
PERMANOVA: permutational multivariate analysis of variance
SCL: San Clemente Island
SCZ: Santa Cruz Island
SMI: San Miguel Island
SNI: San Nicolas Island
SRI: Santa Rosa Island.
