# Supplementary material for: Effect of Geography and Captivity on Scat Bacterial Communities in the Imperiled Channel Island Fox
Source: Front Microbiol. 2021 Dec 1;12:748323. doi: 10.3389/fmicb.2021.748323 (PMC8672056; doi:10.3389/fmicb.2021.748323)
Supplement: Supplementary file 1 [file Data_Sheet_1.docx]

**Adams et al. Supplementary Materials**

**Table S1.** Details for scat samples. Scat samples were collected from all six inhabited islands which included: San Miguel (SMI), Santa Rosa (SRI), Santa Cruz (SCZ), Santa Catalina (CAT), San Clemente (SCL), and San Nicolas (SNI) as well as the Orange County Zoo (OCZ) and the Santa Barbara Zoo (SBZ). Samples were extracted along with extraction blanks (BLANK) and sequenced along with a mock community (MOCK).

| ID | Specimen number | Source | Region | Captivity status | Sex | Age | Body condition | Weight (kg) | Year collected | Month collected |
| --- | --- | --- | --- | --- | --- | --- | --- | --- | --- | --- |
| SMI.4 | 2249 | SMI | north | wild | M | 3 | 2 | 2.29 | 2015 | Jan |
| SMI.5 | 8875 | SMI | north | wild | F |  |  |  | 2015 | Jan |
| SMI.6 | 15754 | SMI | north | wild | F |  |  |  | 2015 | Jan |
| SMI.7 | 15761 | SMI | north | wild | M |  |  |  | 2015 | Jan |
| SMI.8 | 24411 | SMI | north | wild | F |  |  |  | 2015 | Jan |
| SMI.9^*^ | 26235 | SMI | north | wild | M |  |  |  | 2014 | Nov |
| SMI.10 | 77488 | SMI | north | wild | F | 3 | 2 | 1.75 | 2014 | Nov |
| SMI.11 | 87117 | SMI | north | wild | F |  |  |  | 2015 | Jan |
| SMI.12 | 88361 | SMI | north | wild | F | 2 | 2 | 2.3 | 2015 | Jan |
| SMI.13 | 1124C | SMI | north | wild | M | 4 | 3 | 2.68 | 2015 | Jan |
| SMI.14 | 24902-F388 | SMI | north | wild | F | 1 | 2 | 1.9 | 2014 | Dec |
| SMI.15^*^ | C4F60-M281 | SMI | north | wild | M | 2 | 2 | 2.45 | 2014 | Nov |
| SMI.16 | C7602-F348 | SMI | north | wild |  |  |  |  | 2015 | Jan |
| SRI.17 | 15792 | SRI | north | wild | F | 2 | 3 | 1.95 | 2014 | Nov |
| SRI.18 | 15793 | SRI | north | wild | M | 3 | 4 | 2.53 | 2014 | Nov |
| SRI.19^*^ | 16090 | SRI | north | wild | F | 0 | 2 | 1.9 | 2014 | Nov |
| SRI.20^*^ | 16101 | SRI | north | wild | F | 2 | 4 | 2 | 2014 | Nov |
| SRI.21 | 16122 | SRI | north | wild | F | 2 | 2 | 2.01 | 2014 | Nov |
| SRI.22 | 16123 | SRI | north | wild | F | 1 | 4 | 2.6 | 2014 | Nov |
| SRI.23 | 16444 | SRI | north | wild | M | 0 | 3 | 2.17 | 2014 | Dec |
| SRI.24 | 16500 | SRI | north | wild | F | 2 | 4 | 2.37 | 2014 | Dec |
| SRI.25 | 37599 | SRI | north | wild | F | 2 | 4 | 2.21 | 2014 | Nov |
| SRI.26 | 38956 | SRI | north | wild | F | 3 | 2 | 2.07 | 2014 | Nov |
| SRI.27 | 62035 | SRI | north | wild | M | 3 | 3 | 2.3 | 2014 | Dec |
| SRI.28 | 86634 | SRI | north | wild | F | 3 | 3 | 2.31 | 2014 | Dec |
| SRI.29 | 3604E | SRI | north | wild | F | 3 | 3 | 2.28 | 2014 | Dec |
| SCZ.1 | 12846 | SCZ | north | wild | M | 1 | 3 | 1.89 | 2015 | Jan |
| SCZ.2 | 36422 | SCZ | north | wild | M | 2 | 2 | 1.46 | 2015 | Jan |
| SCZ.3 | 51026 | SCZ | north | wild | F | 1 | 3 | 1.71 | 2015 | Jan |
| CAT.50 | 21133 | CAT | south | wild | F | 2 | 3 | 2.61 | 2014 | Oct |
| CAT.51 | 20852 | CAT | south | wild | F | 2 | 2.5 | 2.06 | 2014 | Oct |
| CAT.53 | 63425 | CAT | south | wild | F | 1 | 3 | 2.59 | 2014 | Oct |
| CAT.54 | 95519 | CAT | south | wild | M | 1 | 3 | 2.5 | 2014 | Oct |
| CAT.55 | 45F62 | CAT | south | wild | M | 3 | 5 | 3.36 | 2014 | Oct |
| CAT.57 | 3792D | CAT | south | wild | F | 1 | 4 | 2.07 | 2014 | Nov |
| CAT.58 | B0F74 | CAT | south | wild | M | 1 | 3 | 2.7 | 2014 | Oct |
| CAT.59 | 1477A | CAT | south | wild | M | 3 | 2 | 2.43 | 2014 | Nov |
| CAT.60 | 35E15 | CAT | south | wild | F | 3 | 3 | 2.4 | 2014 | Dec |
| CAT.63 | 97338 | CAT | south | wild | F | 3 | 2 | 1.87 | 2014 | Oct |
| CAT.16C09 | 16C09 | CAT | south | wild | M | 4 | 4.5 | 2.86 | 2014 | Oct |
| CAT.20377 | 20377 | CAT | south | wild | F | 3 | 2 | 1.86 | 2014 | Oct |
| CAT.8383D | 8383D | CAT | south | wild | M | 1 | 3 | 2.5 | 2014 | Oct |
| CAT.E2211^*^ | E2211 | CAT | south | wild | F | 3 | 3 | 2.36 | 2014 | Oct |
| CAT.F0534^*^ | F0534 | CAT | south | wild | M | 1 | 4.5 | 3.08 | 2015 | Oct |
| SCL.1 | 982000190551073 | SCL | south | wild | M | 1 | 3 | 2.07 | 2014 | Oct |
| SCL.2 | 982000190686094 | SCL | south | wild | F | 2 | 2 | 1.56 | 2014 | Nov |
| SCL.3 | 982000167831348 | SCL | south | wild | F | 2 | 3 | 1.91 | 2014 | Oct |
| SCL.4 | 982000363935561 | SCL | south | wild | F | 1 | 2 | 1.76 | 2015 | Jan |
| SCL.5 | 4A0A021174 | SCL | south | wild | M | 2 | 2 | 1.77 | 2015 | Jan |
| SCL.7 | 982000190642413 | SCL | south | wild | F | 1 | 3 | 1.64 | 2014 | Nov |
| SCL.8 | 985121017331033 | SCL | south | wild | F | 2 | 2 | 1.81 | 2014 | Nov |
| SCL.9 | 900236000199222 | SCL | south | wild | F | 1 | 2 | 1.55 | 2014 | Nov |
| SCL.10^*^ | 985121017379629 | SCL | south | wild | M | 1 | 3 | 2.28 | 2015 | Jan |
| SCL.11 | 985120032372226 | SCL | south | wild | M | 3 | 3 | 2.08 | 2014 | Oct |
| SCL.13 | 900236000199213 | SCL | south | wild | M | 1 | 2 | 1.98 | 2015 | Jan |
| SCL.14 | 982000363932112 | SCL | south | wild | F | 1 | 3 | 2.05 | 2014 | Dec |
| SCL.15 | 900236000199202 | SCL | south | wild | F | 1 | 2 | 1.2 | 2014 | Oct |
| SCL.17^*^ | 900236000202580 | SCL | south | wild | F | 1 | 3 | 1.71 | 2014 | Nov |
| SCL.19^*^ | 900118001238833 | SCL | south | wild | F | 1 | 3 | 2.33 | 2014 | Oct |
| SCL.20.2 | 985120032249793 | SCL | south | wild | F | 2 | 2 | 1.56 | 2014 | Nov |
| SNI.2B | 1804 | SNI | south | wild | M | 1 |  |  | 2014 | Aug |
| SNI.4B | 1797 | SNI | south | wild | F | 2 | 2 | 1.55 | 2014 | Aug |
| SNI.6A.mix | 76372 | SNI | south | wild | F | 4 | 1 | 1.29 | 2014 | Sep |
| SNI.7A | 17828 | SNI | south | wild | F | 4 | 1 | 1.39 | 2014 | Aug |
| SNI.9A | 38106 | SNI | south | wild | F | 2 | 2 | 1.69 | 2014 | Sep |
| SNI.10A | 1807 | SNI | south | wild | M | 1 |  |  | 2014 | Sep |
| SNI.11 | 32952 | SNI | south | wild | F | 1 | 2 | 1.56 | 2014 | Sep |
| SNI.12A | 82F61 | SNI | south | wild | F | 4 | 1 | 1.5 | 2014 | Sep |
| SNI.13A | 40749 | SNI | south | wild | M | 2 | 1 | 1.21 | 2014 | Sep |
| SNI.14A | 2609 | SNI | south | wild | F | 1 | 3 | 1.53 | 2014 | Sep |
| SNI.15 | 5141C | SNI | south | wild | F | 1 | 2 | 1.8 | 2014 | Sep |
| SNI.17 | 40530 | SNI | south | wild | M | 3 |  |  | 2014 | Sep |
| SNI.19 | 76917 | SNI | south | wild | F | 3 | 3 | 2.02 | 2014 | Sep |
| SNI.20^*^ | 38057 | SNI | south | wild |  |  |  |  |  |  |
| OCZ.1a^+§^ | 1 | OCZ | mainland | captive | M | 11 |  |  | 2014 | Apr |
| SBZ.1^§^ | 700607 | SBZ | mainland | captive | M | 2 |  |  | 2015 | Jan |
| SBZ.2a^+§^ | 102003 | SBZ | mainland | captive | M | 12 |  |  | 2015 | Jan |
| BLANK.2 | BLANK.2 | BLANK | NA | NA |  |  |  |  |  |  |
| BLANK.7 | BLANK.7 | BLANK | NA | NA |  |  |  |  |  |  |
| BLANK.9 | BLANK.9 | BLANK | NA | NA |  |  |  |  |  |  |
| BLANK.10 | BLANK.10 | BLANK | NA | NA |  |  |  |  |  |  |
| BLANK.11 | BLANK.11 | BLANK | NA | NA |  |  |  |  |  |  |
| MockCom.E | MockCom.E | MOCKCOM | NA | NA |  |  |  |  |  |  |

^*^ Technical replicate IDs: SMI.9.2, SMI.15.2, SRI.19.2, SRI.20.2, CAT.E2211.2, CAT.F0534.2, SCL.10.2, SCL.17.2, SCL.19.2, SNI.20.2

^+^ Biological replicate IDs: OCZ.1b, SBZ.2b

**^§^** Original lab IDs: OCZ1a = OCZOO.1, OCZ.1b = OCZ.2, SBZ.1 = SBZOO.1, SBZ.2a = SBZ.3, SBZ.2b = SBZ.4

**Table S2.** 16S rRNA V4 primer constructs for **A)** forward and **B)** reverse primers.

**A**

| Number | Illumina adapter | Sequencing primer | Ns | Barcode | 16S 515F |
| --- | --- | --- | --- | --- | --- |
| 515F_1 | AATGATACGGCGACCACCGAGATCTACAC | TCTTTCCCTACACGACGCTCTTCCGATCT | NNNN | TCAGC | GTGCCAGCMGCCGCGGTAA |
| 515F_2 | AATGATACGGCGACCACCGAGATCTACAC | TCTTTCCCTACACGACGCTCTTCCGATCT | NNNN | GTATC | GTGCCAGCMGCCGCGGTAA |
| 515F_3 | AATGATACGGCGACCACCGAGATCTACAC | TCTTTCCCTACACGACGCTCTTCCGATCT | NNNN | GCTAC | GTGCCAGCMGCCGCGGTAA |
| 515F_4 | AATGATACGGCGACCACCGAGATCTACAC | TCTTTCCCTACACGACGCTCTTCCGATCT | NNNN | ACGCA | GTGCCAGCMGCCGCGGTAA |
| 515F_5 | AATGATACGGCGACCACCGAGATCTACAC | TCTTTCCCTACACGACGCTCTTCCGATCT | NNNN | GAGAC | GTGCCAGCMGCCGCGGTAA |
| 515F_6 | AATGATACGGCGACCACCGAGATCTACAC | TCTTTCCCTACACGACGCTCTTCCGATCT | NNNN | GACTC | GTGCCAGCMGCCGCGGTAA |
| 515F_7 | AATGATACGGCGACCACCGAGATCTACAC | TCTTTCCCTACACGACGCTCTTCCGATCT | NNNN | CTAGC | GTGCCAGCMGCCGCGGTAA |
| 515F_8 | AATGATACGGCGACCACCGAGATCTACAC | TCTTTCCCTACACGACGCTCTTCCGATCT | NNNN | CGCTC | GTGCCAGCMGCCGCGGTAA |
| 515F_9 | AATGATACGGCGACCACCGAGATCTACAC | TCTTTCCCTACACGACGCTCTTCCGATCT | NNNN | aacat | GTGCCAGCMGCCGCGGTAA |
| 515F_10 | AATGATACGGCGACCACCGAGATCTACAC | TCTTTCCCTACACGACGCTCTTCCGATCT | NNNN | agtgg | GTGCCAGCMGCCGCGGTAA |
| 515F_11 | AATGATACGGCGACCACCGAGATCTACAC | TCTTTCCCTACACGACGCTCTTCCGATCT | NNNN | tgacc | GTGCCAGCMGCCGCGGTAA |
| 515F_12 | AATGATACGGCGACCACCGAGATCTACAC | TCTTTCCCTACACGACGCTCTTCCGATCT | NNNN | tggat | GTGCCAGCMGCCGCGGTAA |
| 515F_13 | AATGATACGGCGACCACCGAGATCTACAC | TCTTTCCCTACACGACGCTCTTCCGATCT | NNNN | tactg | GTGCCAGCMGCCGCGGTAA |
| 515F_14 | AATGATACGGCGACCACCGAGATCTACAC | TCTTTCCCTACACGACGCTCTTCCGATCT | NNNN | cccca | GTGCCAGCMGCCGCGGTAA |

**B**

| Number | Illumina adapter | Index | Sequencing primer | 16S 806R primer |  |
| --- | --- | --- | --- | --- | --- |
| 806R_1 | CAAGCAGAAGACGGCATACGAGAT | ATCACG | GTGACTGGAGTTCAGACGTGTGCTCTTCCGATCT | GGACTACHVGGGTWTCTAAT | |
| 806R_2 | CAAGCAGAAGACGGCATACGAGAT | cgatgt | GTGACTGGAGTTCAGACGTGTGCTCTTCCGATCT | GGACTACHVGGGTWTCTAAT | |
| 806R_3 | CAAGCAGAAGACGGCATACGAGAT | ttaggc | GTGACTGGAGTTCAGACGTGTGCTCTTCCGATCT | GGACTACHVGGGTWTCTAAT | |
| 806R_4 | CAAGCAGAAGACGGCATACGAGAT | tgacca | GTGACTGGAGTTCAGACGTGTGCTCTTCCGATCT | GGACTACHVGGGTWTCTAAT | |
| 806R_5 | CAAGCAGAAGACGGCATACGAGAT | acagtg | GTGACTGGAGTTCAGACGTGTGCTCTTCCGATCT | GGACTACHVGGGTWTCTAAT | |
| 806R_6 | CAAGCAGAAGACGGCATACGAGAT | gccaat | GTGACTGGAGTTCAGACGTGTGCTCTTCCGATCT | GGACTACHVGGGTWTCTAAT | |
| 806R_7 | CAAGCAGAAGACGGCATACGAGAT | cagatc | GTGACTGGAGTTCAGACGTGTGCTCTTCCGATCT | GGACTACHVGGGTWTCTAAT | |
| 806R_8 | CAAGCAGAAGACGGCATACGAGAT | acttga | GTGACTGGAGTTCAGACGTGTGCTCTTCCGATCT | GGACTACHVGGGTWTCTAAT | |
| 806R_9 | CAAGCAGAAGACGGCATACGAGAT | atgtca | GTGACTGGAGTTCAGACGTGTGCTCTTCCGATCT | GGACTACHVGGGTWTCTAAT | |
| 806R_10 | CAAGCAGAAGACGGCATACGAGAT | ccgtcc | GTGACTGGAGTTCAGACGTGTGCTCTTCCGATCT | GGACTACHVGGGTWTCTAAT | |
| 806R_11 | CAAGCAGAAGACGGCATACGAGAT | gtccgc | GTGACTGGAGTTCAGACGTGTGCTCTTCCGATCT | GGACTACHVGGGTWTCTAAT | |
| 806R_12 | CAAGCAGAAGACGGCATACGAGAT | gtgaaa | GTGACTGGAGTTCAGACGTGTGCTCTTCCGATCT | GGACTACHVGGGTWTCTAAT | |
| 806R_13 | CAAGCAGAAGACGGCATACGAGAT | caccgg | GTGACTGGAGTTCAGACGTGTGCTCTTCCGATCT | GGACTACHVGGGTWTCTAAT | |
| 806R_14 | CAAGCAGAAGACGGCATACGAGAT | cacgat | GTGACTGGAGTTCAGACGTGTGCTCTTCCGATCT | GGACTACHVGGGTWTCTAAT | |

**Table S3.** Number of samples (N; excluding the mock community), ASVs, and total target amplicons at each analysis step.

| Step | N | ASVs | Target amplicons |
| --- | --- | --- | --- |
| Start | 94 | 8054 | 9421247 |
| Decontamination | 94 | 8042 | 9283245 |
| Filtering | 89 | 8042 | 9281918 |
| Technical replicates merged | 79 | 8042 | 8231785 |

**Table S5.** Results of the ANOVA and Tukey HSD post hoc tests between sources for differences in relative abundance of recovered ASVs from **(A)** 7 major taxa and **(B)** the top genera. Significant p-values (adjusted for multiple comparisons) are in bold.

**A**

|  | Bacteroidetes | Firmicutes | Fusobacteria | Gammaproteobacteria | Tenericutes | Spirochaetes | Actinobacteria |
| --- | --- | --- | --- | --- | --- | --- | --- |
| Mean % proportion (sd) | 38(12.2) | 30.1(11.6) | 13.2(12) | 8.03(7.53) | 0.653(1.3) | 0.613(1.01) | 0.361(0.67) |
| ANOVA | **0.00895** | **7.05E-09** | **4.07E-06** | **0.0219** | **0.0385** | **0.0352** | **7.30E-15** |
| Tukey HSD comparisons | |  |  |  |  |  |  |
| SRI-SMI | 0.404 | 0.996 | 0.054 | 0.905 | 0.999 | 0.98 | 1 |
| SCZ-SMI | 0.903 | 0.807 | 0.233 | 0.999 | 1 | 1 | 1 |
| CAT-SMI | 1 | **0.002** | **0** | 0.274 | 0.882 | 0.995 | 0.333 |
| SCL-SMI | 1 | 0.113 | **0** | 0.998 | 0.126 | 0.892 | 1 |
| SNI-SMI | 1 | 0.997 | **0.019** | 0.406 | 1 | **0.028** | 0.873 |
| SBZ-SMI | 0.769 | **0.006** | **0.043** | 0.056 | 1 | 1 | 0.998 |
| OCZ-SMI | 0.225 | **0** | 0.476 | 1 | 1 | 1 | **0** |
| SCZ-SRI | 1 | 0.53 | 0.999 | 0.886 | 1 | 0.984 | 1 |
| CAT-SRI | 0.373 | **0** | **0.032** | 0.965 | 0.546 | 1 | 0.202 |
| SCL-SRI | 0.288 | **0.015** | 0.694 | 0.997 | **0.028** | 1 | 1 |
| SNI-SRI | 0.648 | 0.842 | 1 | 0.991 | 0.961 | 0.273 | 0.734 |
| SBZ-SRI | 0.094 | **0.001** | 0.901 | 0.31 | 1 | 0.986 | 0.993 |
| OCZ-SRI | **0.014** | **0** | 1 | 0.98 | 1 | 0.997 | **0** |
| CAT-SCZ | 0.903 | 0.97 | 0.852 | 0.51 | 0.969 | 0.993 | 0.575 |
| SCL-SCZ | 0.872 | 1 | 1 | 0.98 | 0.585 | 0.951 | 1 |
| SNI-SCZ | 0.967 | 0.957 | 1 | 0.597 | 0.999 | 0.28 | 0.881 |
| SBZ-SCZ | 0.346 | 0.601 | 0.999 | 0.101 | 1 | 1 | 0.985 |
| OCZ-SCZ | 0.077 | **0.007** | 1 | 1 | 1 | 1 | **0** |
| SCL-CAT | 1 | 0.772 | 0.68 | 0.62 | 0.833 | 0.999 | 0.163 |
| SNI-CAT | 1 | **0.013** | 0.065 | 1 | 0.99 | 0.144 | 0.986 |
| SBZ-CAT | 0.748 | 0.879 | 0.998 | 0.688 | 0.942 | 0.994 | 0.996 |
| OCZ-CAT | 0.21 | **0.009** | 0.921 | 0.816 | 0.973 | 0.999 | **0** |
| SNI-SCL | 0.999 | 0.41 | 0.859 | 0.77 | 0.323 | 0.38 | 0.703 |
| SBZ-SCL | 0.785 | 0.34 | 1 | 0.128 | 0.499 | 0.956 | 0.993 |
| OCZ-SCL | 0.233 | **0.001** | 1 | 0.998 | 0.689 | 0.988 | **0** |
| SBZ-SNI | 0.605 | **0.021** | 0.954 | 0.617 | 0.998 | 0.29 | 1 |
| OCZ-SNI | 0.142 | **0** | 1 | 0.866 | 0.999 | 0.547 | **0** |
| OCZ-SBZ | 0.979 | 0.396 | 0.999 | 0.259 | 1 | 1 | **0** |

**B**

|  | Bacteroides | Fusobacterium | Escherichia | Cetobacterium | Clostridium | Streptococcus |
| --- | --- | --- | --- | --- | --- | --- |
| Mean % proportion (sd) | 27.6(9.85) | 10.8(8.11) | 2.79(5.38) | 2.41(9.97) | 1.75(2.82) | 0.631(3.77) |
| ANOVA | **0.0233** | **0.00112** | **0.00828** | **0.000614** | **5.66E-06** | **4.94E-09** |
| Tukey HSD comparisons | |  |  |  |  |  |
| SRI-SMI | 0.404 | 0.981 | 0.969 | **0.002** | 0.101 | 1.000 |
| SCZ-SMI | 0.401 | 1.000 | 1.000 | 0.181 | 0.689 | 1.000 |
| CAT-SMI | 1.000 | **0.018** | 0.104 | **0.001** | 0.359 | 0.977 |
| SCL-SMI | 1.000 | 0.881 | 1.000 | **0.001** | 0.139 | 1.000 |
| SNI-SMI | 0.986 | 0.999 | 0.994 | **0.001** | 0.075 | 1.000 |
| SBZ-SMI | 0.981 | 0.938 | 1.000 | 0.181 | 0.976 | 1.000 |
| OCZ-SMI | 0.691 | 1.000 | 0.999 | 0.380 | **0.001** | **2.108E-10** |
| SCZ-SRI | 0.991 | 0.995 | 1.000 | 1.000 | 1.000 | 1.000 |
| CAT-SRI | 0.399 | **0.001** | **0.005** | 1.000 | 0.996 | 0.977 |
| SCL-SRI | 0.365 | 0.297 | 0.980 | 1.000 | 1.000 | 1.000 |
| SNI-SRI | 0.904 | 1.000 | 1.000 | 1.000 | 1.000 | 1.000 |
| SBZ-SRI | 0.339 | 0.661 | 0.993 | 1.000 | 0.995 | 1.000 |
| OCZ-SRI | 0.112 | 0.999 | 1.000 | 1.000 | **3.923E-06** | **2.066E-10** |
| CAT-SCZ | 0.409 | 0.554 | 0.413 | 1.000 | 1.000 | 0.999 |
| SCL-SCZ | 0.393 | 0.999 | 1.000 | 1.000 | 1.000 | 1.000 |
| SNI-SCZ | 0.742 | 1.000 | 1.000 | 1.000 | 1.000 | 1.000 |
| SBZ-SCZ | 0.252 | 0.994 | 1.000 | 1.000 | 0.999 | 1.000 |
| OCZ-SCZ | 0.086 | 1.000 | 1.000 | 1.000 | **1.983E-04** | **9.225E-08** |
| SCL-CAT | 1.000 | 0.318 | **0.051** | 1.000 | 1.000 | 0.969 |
| SNI-CAT | 0.989 | **0.003** | **0.010** | 1.000 | 0.992 | 0.975 |
| SBZ-CAT | 0.975 | 0.985 | 0.794 | 1.000 | 1.000 | 0.999 |
| OCZ-CAT | 0.660 | 0.711 | 0.489 | 1.000 | **1.377E-05** | **1.589E-09** |
| SNI-SCL | 0.985 | 0.544 | 0.997 | 1.000 | 1.000 | 1.000 |
| SBZ-SCL | 0.976 | 1.000 | 1.000 | 1.000 | 0.999 | 1.000 |
| OCZ-SCL | 0.664 | 0.999 | 1.000 | 1.000 | **5.111E-06** | **1.065E-10** |
| SBZ-SNI | 0.813 | 0.808 | 0.998 | 1.000 | 0.992 | 1.000 |
| OCZ-SNI | 0.389 | 1.000 | 1.000 | 1.000 | **3.031E-06** | **1.688E-10** |
| OCZ-SBZ | 0.995 | 0.995 | 0.999 | 1.000 | **0.001** | **9.046E-08** |

**Table S6.** Dispersion tests based on Bray-Curtis dissimilarity. Factors with homogenous dispersions are in bold.

|  | Df | sumSq | meanSq | F | P |
| --- | --- | --- | --- | --- | --- |
| Islands (N = 62) |  |  |  |  |  |
| Island | 5 | 0 | 0.03 | 6.5 | 7.60E-05 |
| Sex | 1 | 0 | 0.01 | 6.1 | 1.64E-02 |
| Condition | 6 | 1 | 0.09 | 20.0 | 0 |
| Age | 4 | 0 | 0.02 | 7.2 | 8.69e-05 |
| Year collected | 1 | 0 | 0.02 | 12.0 | 1.12E-03 |
| Month collected | 5 | 0 | 0.03 | 6.4 | 9.70E-05 |
| Weight | 52 | 2 | 0.03 | 87.0 | 0 |
| Extracted group | 13 | 1 | 0.04 | 6.2 | 1.30E-06 |
| SMI (N = 6) |  |  |  |  |  |
| Sex | 1 | 0 | 0.00 | 0.18 | **0.697** |
| Condition | 1 | 0 | 0.08 | 100 | 5.33E-04 |
| Age | 3 | 0 | 0.02 | 6.8E30 | 1.48E-31 |
| Year collected | 1 | 0 | 0.00 | 7.0e-03 | **0.938** |
| Month collected | 2 | 0 | 0.03 | 4.0 | **0.141** |
| Weight | 5 | 0 | 0 | - | - |
| Extracted group | 2 | 0 | 0.05 | 18 | 0.0211 |
| SRI (N = 13) |  |  |  |  |  |
| Sex | 1 | 0 | 0.01 | 0.93 | **0.356** |
| Condition | 2 | 0 | 0.01 | 0.62 | **0.559** |
| Age | 3 | 0 | 0.04 | 4.40 | 0.0366 |
| Month collected | 1 | 0 | 0.02 | 3.00 | **0.111** |
| Weight | 12 | 0 | 0 | - | - |
| Extracted group | 2 | 0 | 0.06 | 8.30 | 0.00758 |
| CAT (N = 15) |  |  |  |  |  |
| Sex | 1 | 0 | 0.00 | 2.7e-01 | **0.611** |
| Condition | 5 | 0 | 0.08 | 100 | 1.00E-07 |
| Age | 3 | 0 | 0.06 | 37 | 4.80E-06 |
| Year collected | 1 | 0 | 0.17 | 88 | 4.00E-07 |
| Month collected | 2 | 0 | 0.09 | 40 | 4.60E-06 |
| Weight | 13 | 0 | 0.02 | 3.0E30 | 0 |
| Extracted group | 2 | 0 | 0.08 | 30 | 2.12E-05 |
| SCL (N = 15) |  |  |  |  |  |
| Sex | 1 | 0 | 0.00 | 0.044 | **0.837** |
| Condition | 1 | 0 | 0.00 | 0.0081 | **0.93** |
| Age | 2 | 0 | 0.09 | 37.0 | 7.60E-06 |
| Year collected | 1 | 0 | 0.00 | 0.14 | **0.71** |
| Month collected | 3 | 0 | 0.04 | 4.9 | 2.06E-02 |
| Weight | 14 | 0 | 0 | - | - |
| Extracted group | 2 | 0 | 0.01 | 0.68 | **0.525** |
| SNI (N = 10) |  |  |  |  |  |
| Sex | 1 | 0 | 0.08 | 51.0 | 9.81E-05 |
| Condition | 2 | 0 | 0.01 | 1.3 | **0.328** |
| Age | 3 | 0 | 0.03 | 2.7 | **0.139** |
| Month collected | 1 | 0 | 0.02 | 3.0 | **0.122** |
| Weight | 9 | 0 | 0 | - | - |
| Extracted group | 3 | 0 | 0.05 | 4.9 | 4.68E-02 |
| Captivity (N = 21) |  |  |  |  |  |
| Source | 2 | 0 | 0.07 | 9.40 | 1.57E-03 |
| Sex | 1 | 0 | 0.01 | 2.30 | **0.149** |
| Age | 4 | 0 | 0.08 | 23.00 | 1.60E-06 |
| Year Collected | 1 | 0 | 0.00 | 0.27 | **0.611** |
| Month Collected | 4 | 0 | 0.04 | 9.20 | 4.80E-04 |
| Wild vs Captive | 1 | 0 | 0.00 | 0.49 | **0.491** |
| Wild or Captive born | 1 | 0 | 0.01 | 0.70 | **0.414** |

Df = degrees of freedom, sumSq = sum of squares, meanSq = mean squares, F = F-statistic, P = p-value.

**Table S7**. Top differentially abundant genera for significant comparisons. The values in parentheses are the total number of differentially abundant genera at a significance level of α=0.01 for that comparison. Adjusted p-values are reported from a Wald test.

| Comparison | ASV | log2Fold  Change | lfcSE | padj | Genera |
| --- | --- | --- | --- | --- | --- |
| SRI | |  |  |  |  |
| Nov (2) | a2ccb224ef49d37772c2f224b1811b3b | 7.1 | 1.74 | 1.04E-3 | *Bilophila* |
| Dec (1) | efcd0461ac1ed0fea763be1234b924d9 | -26.2 | 3.05 | 5.91E-16 | *Megamonas* |
| CAT | |  |  |  |  |
| Female (1) | 93c484472e6e5c4e1afecbc0c2a50375 | 3.0 | 0.78 | 5.8E-3 | *Acinetobacter* |
| Male (1) | 5d1a7a11e80c627fbc98743087f309f0 | -4.12 | 0.90 | 3.86E-4 | *Roseburia* |
| SCL | |  |  |  |  |
| 2014 (3) | c003202be6a53b07fbb26ecd2161fe2c | 22.4 | 3.09 | 1.79E-11 | *Lactobacillus* |
| 2015 (1) | efcd0461ac1ed0fea763be1234b924d9 | -27.6 | 3.32 | 7.12E-15 | *Megamonas* |
| Low weight (0) | - | - | - | - | *-* |
| High weight (1) | 82d7750731e70fb003d8c81d683deee7 | -6.71 | 1.46 | 4.31E-4 | *Coprococcus* |
| Captivity |  |  |  |  |  |
| Wild (14) | efcd0461ac1ed0fea763be1234b924d9 | 22.8 | 3.51 | 1.98E-9 | *Megamonas* |
| Captive (1) | ddb43929969b5142b0fd746086cd5afe | -2.16 | 0.67 | 8.07E-3 | *Blautia* |
| 2014 (3) | eee9bab26577ab7978a3c7bb4a082922 | 23.1 | 2.63 | 6.55E-17 | *Bifidobacterium* |
| 2015 (1) | efcd0461ac1ed0fea763be1234b924d9 | -27.3 | 3.11 | 6.55E-17 | *Megamonas* |
| Early months (1) | 8ebdf280ad63688af237441aa2db40d1 | 1.05 | 0.11 | 9.2E-20 | *Rikenella* |
| Late months (2) | efcd0461ac1ed0fea763be1234b924d9 | -1.12 | 0.31 | 0 | *Megamonas* |

**
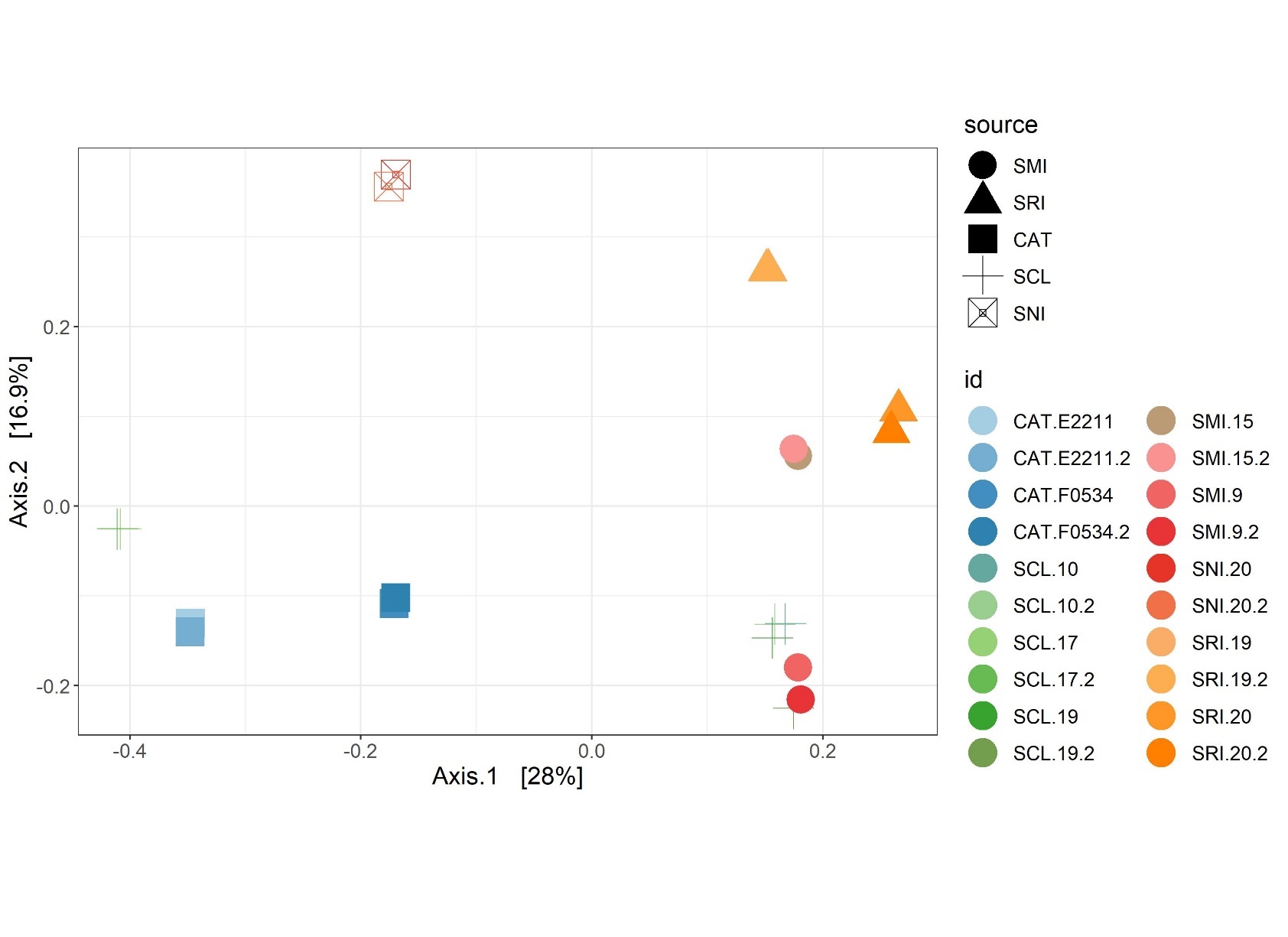
**

**Figure S1.** Principal coordinates analysis (PCoA) based on Bray-Curtis dissimilarity showing technical replicates that were merged for later analyses.

**
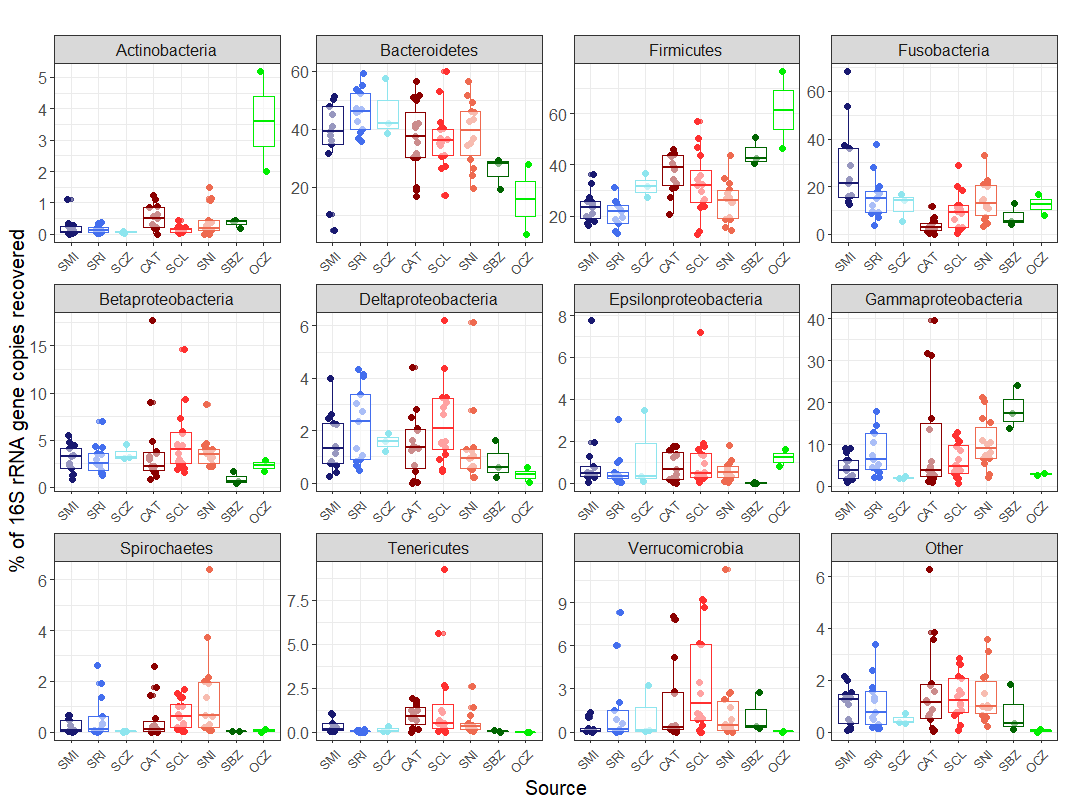
**

**Figure S2.** Boxplots of major taxa compared by source. Note the y-axis is free to vary according to the proportions of each taxon.

**
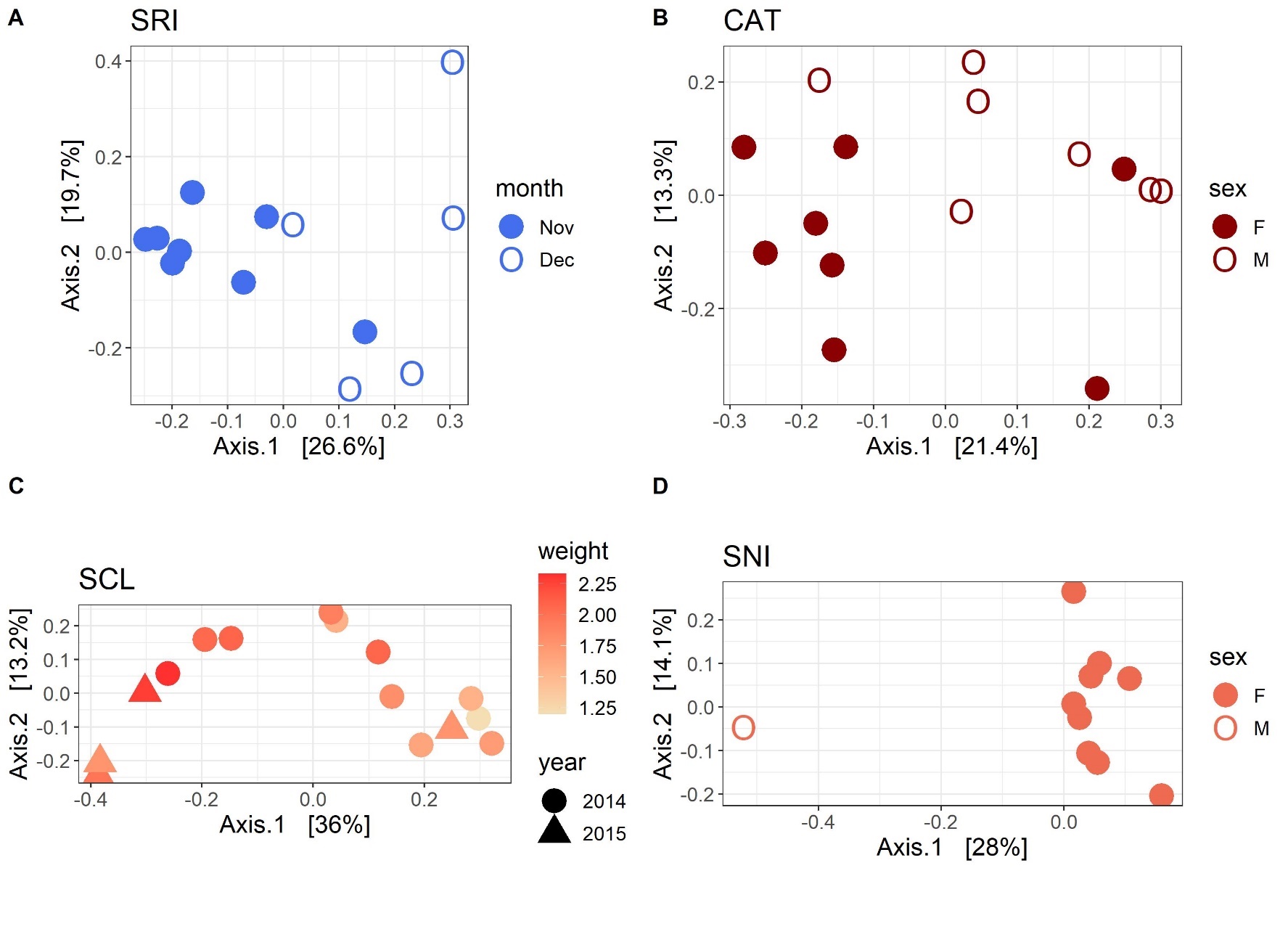
**

**Figure S3.** Principal coordinate analyses for significant factors including **(A)** month that the scat was collected on SRI, **(B)** sex on CAT, **(C)** year the scat was collected and fox weight on SCL, and **(D)** sex on SNI.


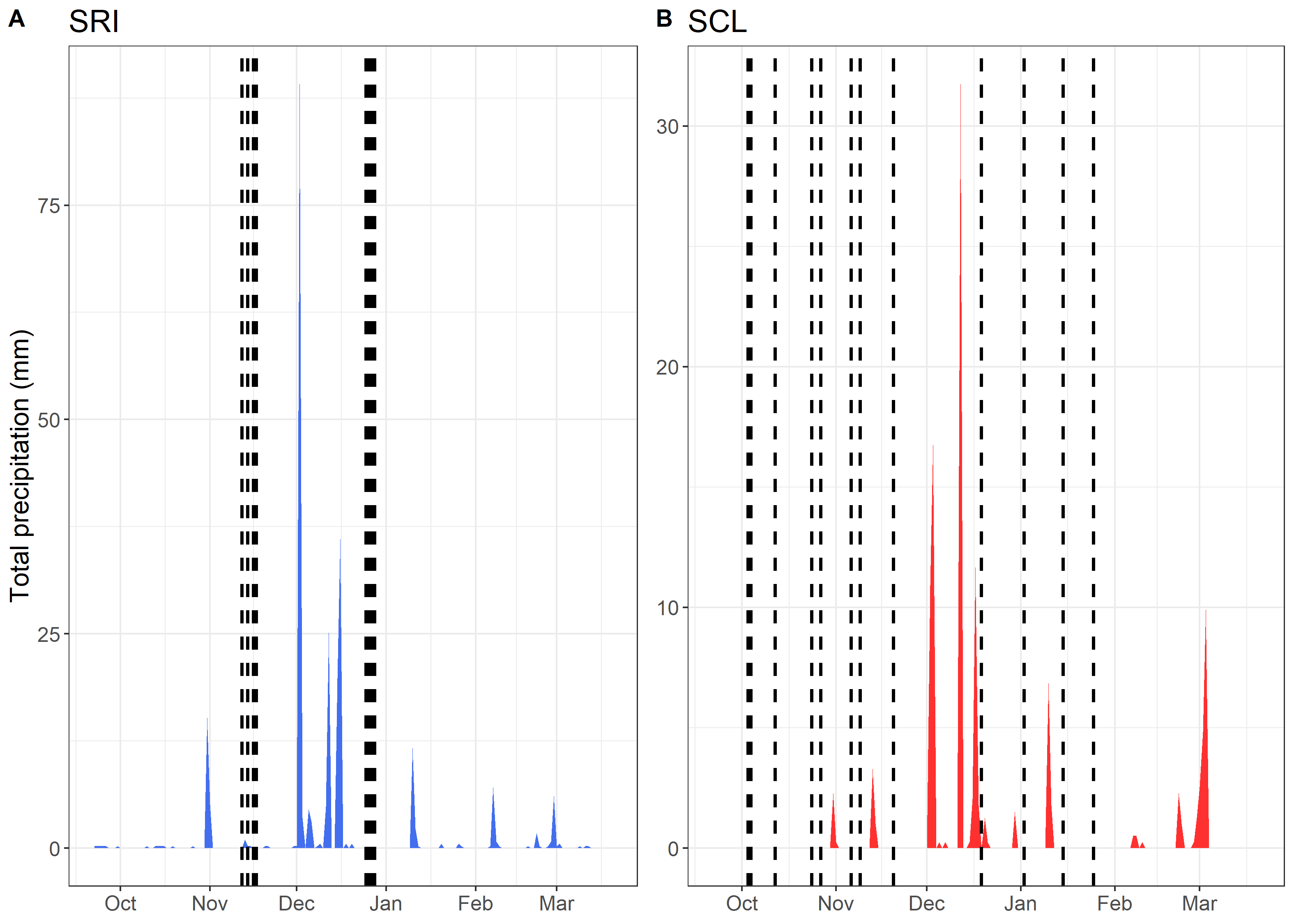


**Figure S4.** The total precipitation from October 2014 to March 2015 with the dates of sample collection indicated with vertical dashed lines for **(A)** SRI and **(B)** SCL.

**
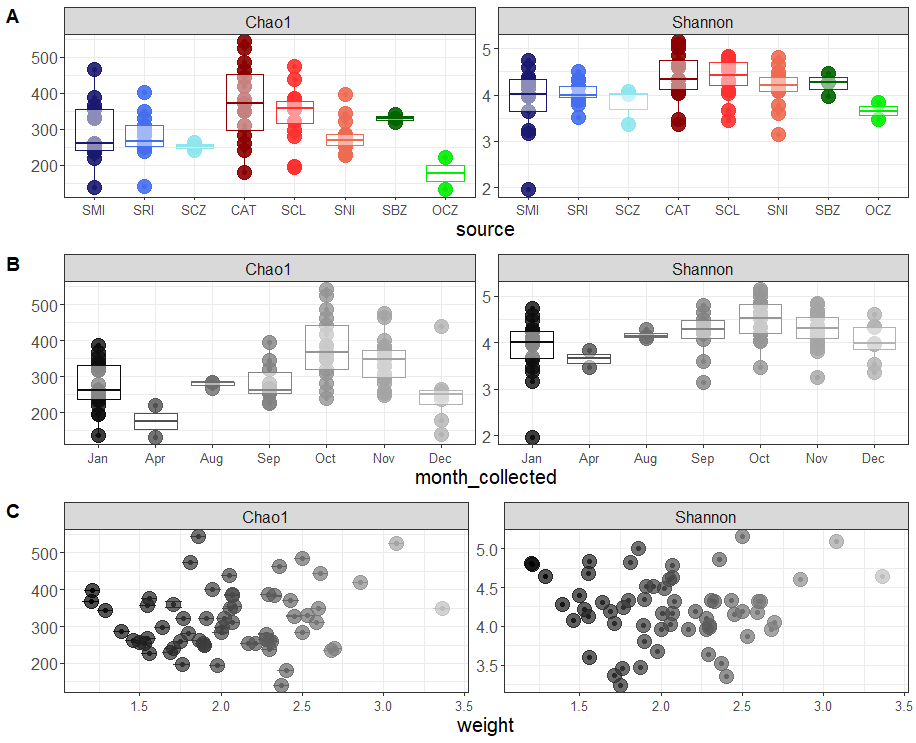
**

**
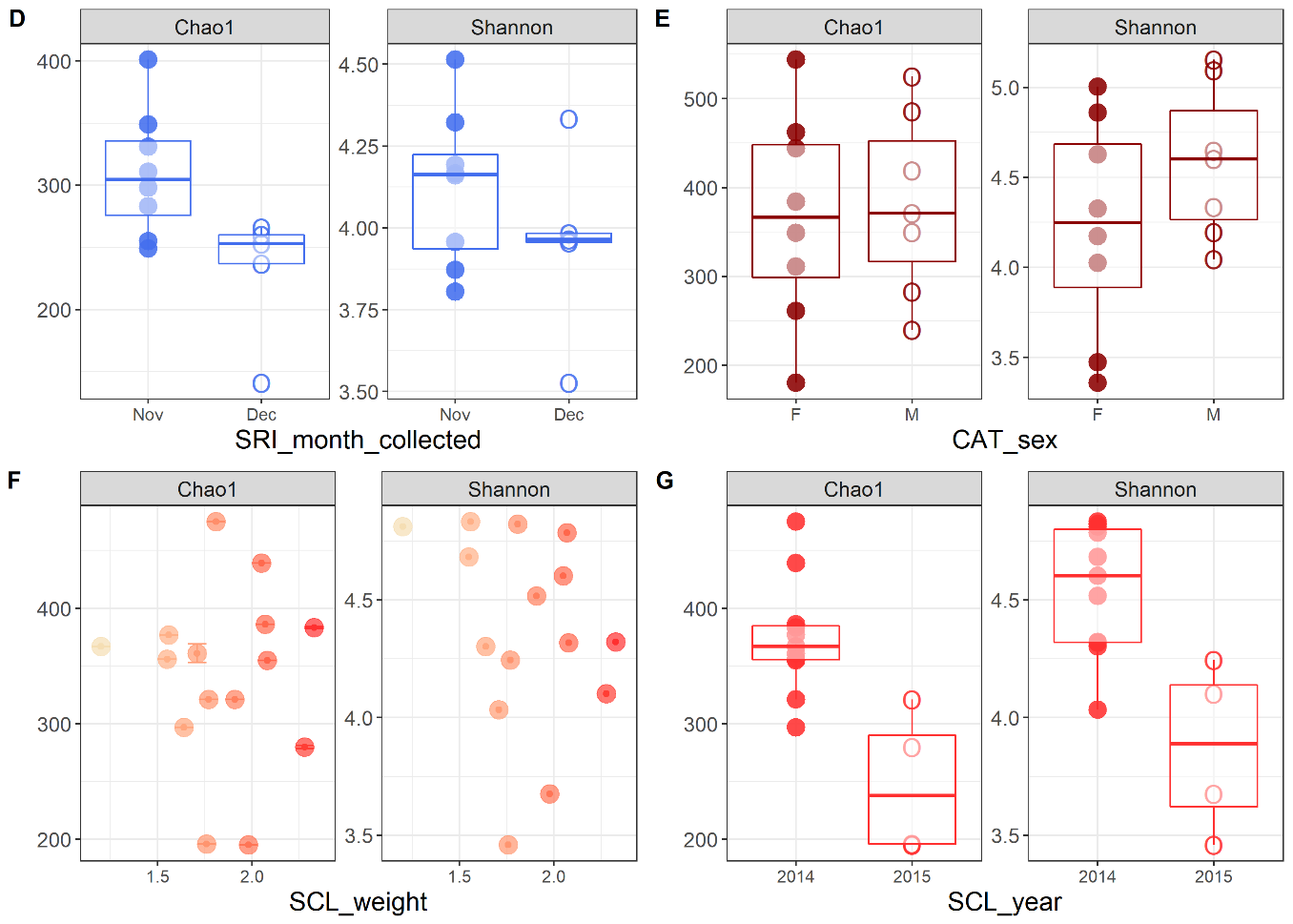
**

**Figure S5.** **(A-C)** Alpha diversity (Chao1 richness and Shannon diversity indexes) between sources, the month the samples were collected, and weight. Samples with missing data were kept for A. **(D-G)** Chao 1 and Shannon diversity metrics for significant factors identified by PERMANOVA tests within an island.
